# Inter-Subunit Dynamics Controls Tunnel Formation During the Oxygenation Process in Hemocyanin Hexamers

**DOI:** 10.3389/fmolb.2021.710623

**Published:** 2021-09-15

**Authors:** Khair Bux, Xiayu Shen, Muhammad Tariq, Junqi Yin, Syed Tarique Moin, Debsindhu Bhowmik, Shozeb Haider

**Affiliations:** ^1^ Third World Center for Science and Technology, H.E.J. Research Institute of Chemistry, International Center for Chemical and Biological Sciences, University of Karachi, Karachi, Pakistan; ^2^ UCL School of Pharmacy, London, United Kingdom; ^3^ Oak Ridge National Laboratory, Center for Computational Sciences, Oak Ridge, TN, United States; ^4^ Oak Ridge National Laboratory, Computer Sciences and Engineering Division, Oak Ridge, TN, United States

**Keywords:** hemocyanin, molecular dynamics, deep learning, metalloprotein, copper

## Abstract

Hemocyanin from horseshoe crab in its active form is a homo-hexameric protein. It exists in open and closed conformations when transitioning between deoxygenated and oxygenated states. Here, we present a detailed dynamic atomistic investigation of the oxygenated and deoxygenated states of the hexameric hemocyanin using explicit solvent molecular dynamics simulations. We focus on the variation in solvent cavities and the formation of tunnels in the two conformational states. By employing principal component analysis and CVAE-based deep learning, we are able to differentiate between the dynamics of the deoxy- and oxygenated states of hemocyanin. Finally, our results identify the deoxygenated open conformation, which adopts a stable, closed conformation after the oxygenation process.

## Introduction

Hemocyanins (HCNs) are large type-3 metalloproteins that transport oxygen in the blood of invertebrates like arthropods and molluscs (Van Holde and Miller, 1995; Terwilliger, 1998; Burmester, 2002; Pick et al., 2008; Rehm et al., 2012; Marxen et al., 2014; Pinnow et al., 2016). Their binuclear active site coordinates copper as metal and accommodates oxygen as a peroxide ion, due to which copper oxidizes from (I) to (II) oxidation state (Himmelwright et al., 1978, 1980; Magnus and Ton-That, 1991; Jaenicke et al., 2012). Besides oxygen transportation, they have also been found to play a major role in diverse physiological functions like homeostasis, transportation of hormones (Prosser and Brown, 1961), in the development of immunity *via* activation of phenoloxidases protein that plays a major role in the formation of their exoskeleton formation and melamine pigments, as well as wound recovery (Lei et al., 2008; Coates et al., 2011; Coates and Nairn, 2014; Zhong et al., 2016; Coates and Talbot, 2018). In addition, HCNs also possess microbicidal property by enhancing the production of reactive oxidative (ROs) species that can develop an immediate defense mechanism against microbes. Furthermore, HCNs have also been reported to have antibacterial and antifungal properties (Dolashka et al., 2016; Qin et al., 2018). The Horseshoe crab (*Limulus polyphemus*) blue blood is being increasingly used in biotechnology and biomedical applications (Gibson III and Hilly, 1992; Riggs et al., 2002; Del Campo et al., 2011; Arancibia et al., 2012; Coates and Nairn, 2014; Gesheva et al., 2014; Kumar et al., 2015; Zhong et al., 2016; Besser et al., 2018). The copper rich blood contains amebocytes, which have an analogous role to the white blood cells in humans and are involved in the defense against pathogens. Thus, blue blood is used to produce Limulus amebocyte lysate (LAL), which has the potential to dysregulate the pathogenicity and virulence effects of pathogenic bacteria by reacting with their endotoxins that cause severe infections in the circulatory systems of humans. Moreover, HCNs have also been found to boost up the immune system of mammals by inducing the potent Th1-dominant immune response (Hanyecz et al., 2004). This property is being exploited in their use as immune stimulants and immune-modulators in cancer (Riggs et al., 2002; Matsushima et al., 2003; Arancibia et al., 2012; Becker et al., 2014; Gesheva et al., 2014; Zhong et al., 2016; Guncheva et al., 2019; Mora Román et al., 2019). More specifically, HCNs from *Megathura crenulata* (keyhole limpet hemocyanin) have shown anti-cancer properties in murine models of colon carcinoma, while being used as immuno-stimulants (Harris and Markl, 1999; Musselli et al., 2001; Miles et al., 2011). In addition, blue blood is widely used in the pharmaceutical industry to control the contamination of their products and medicines and in the development of antibodies and vaccines (Fellers and Harris, 1940; Levin and Bang, 1968; Levin et al., 1970; Berkson and Shuster Jr, 1999; Rutecki et al., 2004; Odell et al., 2005; Mazzotti et al., 2007; Bayer, 2016; Krisfalusi-Gannon et al., 2018; Maloney et al., 2018; Owings et al., 2019).

**FIGURE 1:**
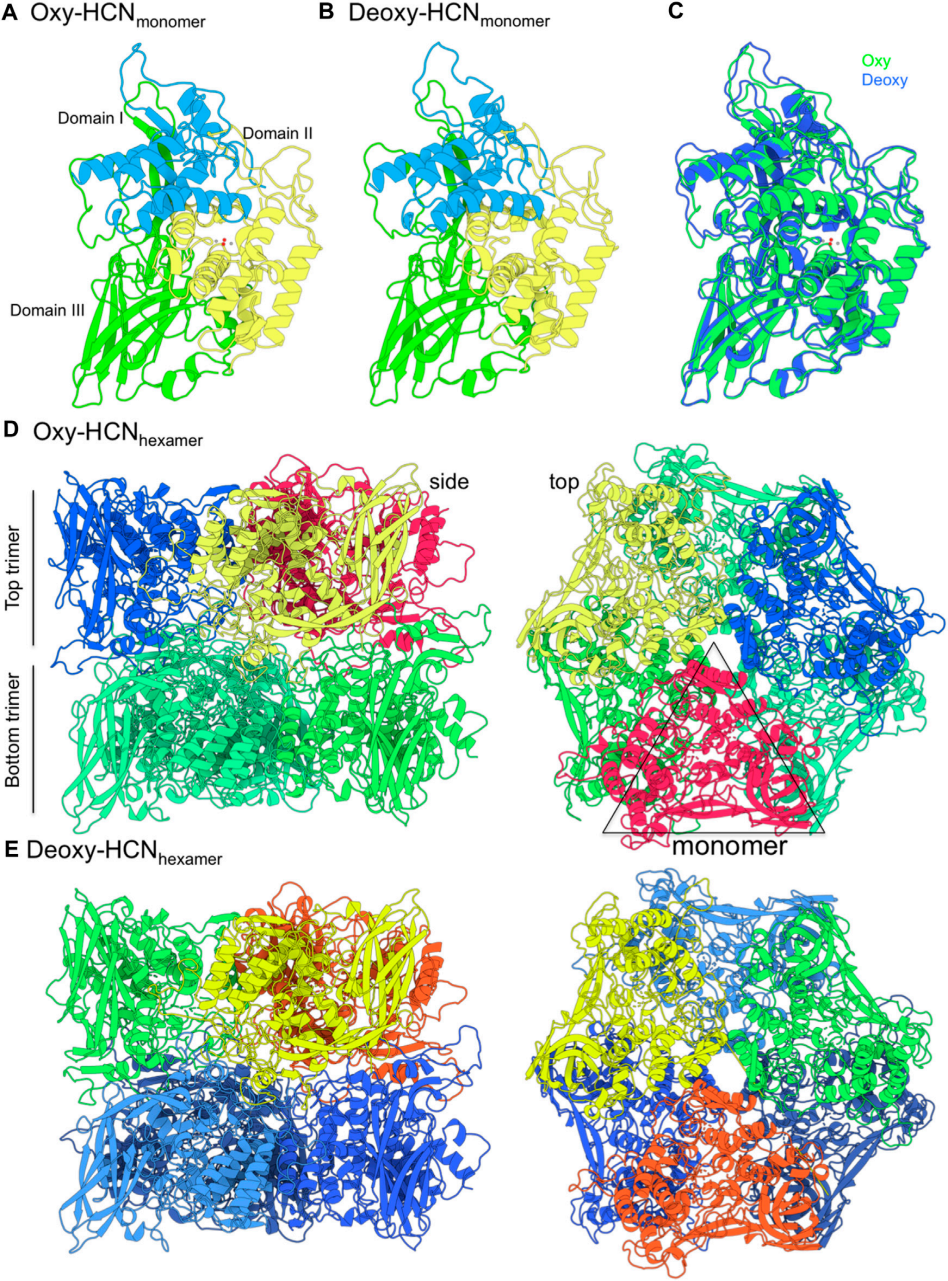
The structure of hemocyanin (HCN) from *Limulus polyphemus* in oxygenated (Oxy) and deoxygenated (Deoxy) states. **(A,B)** The structure is made up of six identical monomers. Each monomer consists of three domains. Domain II contains the copper binding site (grey spheres) that coordinates oxygen atoms (red spheres). **(C)** The monomers from Oxy and Deoxy states differ primarily in the conformation of the loops. **(D,E)** The structure is composed of two concentric layers of homotrimers and arranged as a “dimer-of-trimers.” Monomer subunits in each top trimer are represented in a different color. The bottom trimer units are colored in green (Oxy-HCN) and blue (Deoxy-HCN).

**FIGURE 2:**
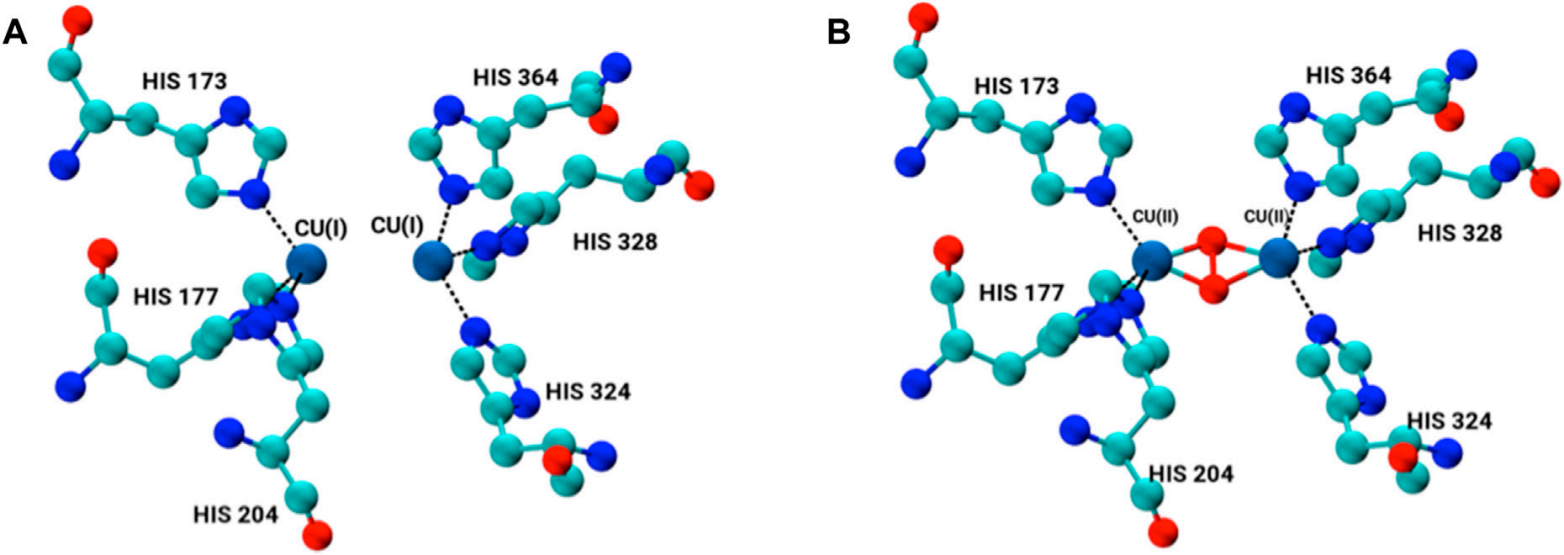
Binuclear copper-containing metal binding site in **(A)** deoxygenated form and **(B)** oxygenated form of each monomer subunit within the hexamer.

The X-ray structure of arthropod HCN is homo-hexameric (Figure 1). Two concentric rings, each consisting of three identical subunits, sit on top of another forming a dimer-of-trimers (Hazes et al., 1993; Magnus et al., 1994). Each subunit consists of three subdomains; 1) the C-terminal domain consists of residues 1 to 154; 2) the N-terminal domain contains residues from 380 to 628; and 3) the metal binding domain that lies between N- and C-terminal domains contains residues ranging from 155 to 379 along with binuclear copper-containing active site (Volbeda and Hol, 1989; Hu et al., 2000; Hundahl et al., 2003; Matsushima et al., 2003). Each copper (I) atom forms a coordination bond with ε nitrogen of three histidine residues, namely, His173, His177, His204 and His324, His328, His364. This represents the deoxygenated form of HCN (Deoxy-HCN). In the oxygenated form (Oxy-HCN) copper reversibly binds with oxygen resulting in the formation of the dicopper peroxo complex (Figure 2). There is subsequent oxidation of copper from Cu(I) to Cu(II) while retaining the coordination between three histidine residues and copper as observed in Deoxy-HCN (Decker and Tuczek, 2000; Hu et al., 2000).

The oxygenation mechanism of multimeric HCN is a complex phenomenon. Previous studies have reported on the detailed role of water in the oxygenation process, including its key role as an allosteric modulator of oxygen accessibility to the binuclear copper site of HCN (Hazes et al., 1993; Hundahl et al., 2003). Some studies have also reported that water in close proximity to copper (II) can also play an intimate role by replacing dioxygen molecule at the active site of HCN (Karplus and McCammon, 2002; Naresh et al., 2015; Bux et al., 2018). Furthermore, the formation of large solvent cavities at the interface of subunits has also been reported. It is *via* these cavities that the movement of oxygen up to the active site is possible (Magnus et al., 1994). The oxygenation process is also allosterically regulated such that the HCN structure acquires different conformational states (higher and lower oxygen affinity states) to attract oxygen to the active site. The allosteric changes are further dependent upon the dynamic behavior of protein that is entirely orchestrated by inter-residual dynamics of the subunits (Volbeda and Hol, 1989; Hazes et al., 1993; Magnus et al., 1994). HCNs have a negatively charged electrostatic surface, which makes solubility and subsequent hydration of HCNs in water possible (Coates and Nairn, 2013). A consequence of the hydration phenomenon is that it prevents the association of HCNs with other negatively charged entities that may affect the physiological function of the protein (Coates and Nairn, 2013).

The biological function of large biomolecules like HCN is dependent upon its conformational arrangement and cannot be explained solely on its structural description. Investigating the dynamic behavior of large biomolecules is, therefore, a key factor to understanding function. Experimental methods like X-ray crystallography are of great value in structural elucidation, but they are quite restricted in exploring the dynamical behavior because of its limitation in time and space resolution (Ohtaki and Radnai, 1993; Ma, 2005; Neutze, 2014). Theoretical and computational methods like molecular dynamics simulations (MD), on other hand, have emerged as the most reliable method to evaluate both structural and dynamical features of biomolecules (Sagui and Darden, 1999; Karplus and McCammon, 2002). In addition, conformational changes that mediate the oxygenation of HCN are entirely a dynamics dependent phenomenon. Therefore, a method that could provide microscopic insights into the dynamic behavior of the HCNs and variations as a function of time needs to be employed.

Many experimental studies have been reported for HCNs (Decker et al., 1988; Volbeda and Hol, 1989; Magnus and Ton-That, 1991; Hazes et al., 1993; Magnus et al., 1994; Jaenicke et al., 2012; Saito and Thiel, 2014; Naresh et al., 2015, 2015; Bux et al., 2018). Most of the up-to-date work commonly discusses the structural features of protein in its monomeric form and rarely in its biologically active hexameric form (Volbeda and Hol, 1989). Very few theoretical and computational studies have been reported for HCNs. Recently, in a combined QM/MM approach, the reversible binding of dioxygen at the binuclear copper active site was reported (Fariselli et al., 1999; Saito and Thiel, 2014). The study mainly focused on the active site of the protein and provides little insight into the structural dynamics of the multimeric protein. A previous study used MD simulations to report insights into the oxygenation mechanism of HCNs and the role of solvent *via* tunnel formation with the help of neighboring amino acid residues of the metal site (Bux et al., 2018). Again, the study was conducted using just one single subunit of HCN instead of the biologically active hexameric form.

Here, we present a detailed atomistic investigation of the hexameric HCN using MD simulations, with a focus on the conformations that the HCN adopts in oxygenated and deoxygenated states. By following the variation in solvent cavities and tunnels, we are able to identify residues that are involved in tunnel formation in Deoxy- and Oxy-HCN. Finally, our principal component analysis and CVAE-based deep learning results are able to differentiate between the global dynamics of these complex systems.

## Methodology

### Modeling of the Hexamer

The biologically active state of *Limulus polyphemus* (Atlantic horseshoe crab) HCN is homo-hexameric. Oxygenated (PDB id 1OXY; Hazes et al., 1993) and deoxygenated (PDB id 1LLA; Magnus et al., 1994) monomeric forms of HCN are available, while the hexameric form is yet to be reported. There are several missing loop residues in the monomeric form. Therefore, we first built the complete monomeric form using the *Panulirus interruptus* (California spiny lobster) structure as a template (PDB id 1HC1; Volbeda and Hol, 1989), which displays 62% sequence homology. The modeled monomeric HCNs were then used to build a hexameric structure by structurally superimposing each subunit from horseshoe crab on hexameric spiny lobster structure. The loops were modeled using Modeller 9v23 (Webb and Sali, 2016) implemented in the Chimera suite (Pettersen et al., 2004).

### Active Site Modeling

The active site of HCN is composed of two binuclear copper atoms each of which is coordinated to three histidine amino acid residues His172, His177, His204 and His324, His328, His 364. As a result of the bond between copper atoms and ε nitrogen NE of the three histidine residues, the geometry of the copper coordination site becomes tetrahedral. The copper adopts oxidation state one Cu(I) in the deoxygenated form of the protein (Magnus and Ton-That, 1991; Hazes et al., 1993; Magnus et al., 1994; Fariselli et al., 1999). In the oxygenated form, the geometry of the binding site changes from tetrahedral to trigonal pyramidal. The oxidation state of copper changes from Cu(I) to Cu(II) upon binding of oxygen as a peroxide. In the present study, the active site in each subunit was modeled by constructing a bond between two copper atoms and the ε-NE atom in the imidazole rings of each six histidine residues, three on each side, in its both deoxygenated and oxygenated form (Figure 2).

### Force Field Parameterization of Copper Cluster

The force field parameters for the copper-containing binuclear active site of the hexamer were constructed for the AMBER ff14SB force field using the bonded approach (Wang et al., 2004; Hornak et al., 2006). The bonded parameters for tetrahedral geometry for Cu(I) in deoxygenated and trigonal bi-pyramidal coordination for Cu(II) in an oxygenated state were taken from our previously reported study (Bux et al., 2018). Since HCNs are homo-hexameric, all six subunits contain the same residues in both deoxygenated and oxygenated states.

### Molecular Dynamics Simulations

The ionization states of the amino acid side chains were determined at pH7.0, using propKa implemented in the play molecule (Martínez-Rosell et al., 2017). The system was described using the ff14SB force field and further processed to run MD simulations with Desmond 3.6 (Bowers et al., 2006). The overall charge was neutralized by manual addition of 150 Na + ions to both systems that were subsequently solvated by adding TIP3P water molecules. In total, 77,896 (Deoxy) and 77,848 (Oxy) water molecules were present in a solvation box with edges set at least 12 Å from the solute atom.

Equilibration of the hydrated systems was then carried out in an NPT ensemble for 50 ns. Using the last frame from the equilibration, the production step was run in the NVT ensemble for both Oxy- and Deoxy-HCN systems. The SHAKE algorithm was used to simulate all bonds of hydrogen atoms in rigid and constrained form (Kräutler et al., 2001). Short bonded and nonbonded interactions were treated using RESPA integrator at the average time interval of about 2 fs (Tuckerman et al., 1992) but longer time step of about 4 fs to simulate the long range interactions using Particle Mesh Ewald algorithms (Cerutti et al., 2009). The production step was run as 10 replicates of 100 ns each for both systems.

### Structural Analysis

The trajectories were visualized using VMD (Humphrey et al., 1996). All analysis was carried out using GROMACS tools (Abraham et al., 2015) and pytraj (Roe and Cheatham, 2013) on the last 40 ns of each replicate, which was considered equilibrated after conventional RMSD analysis. The RMSD analysis was carried out using MDLovofit (Martínez, 2015). The radius of gyration (Rg) was used to assess the compactness of the hexameric structure. The structural figures were generated in VMD (Humphrey et al., 1996) and Protein Imager (Tomasello et al., 2020).

### Dynamics Cross-Correlation Matrixes (DCCM) Analysis

The dynamic behavior of hexameric HCN is a function of the correlated motions of individual subunits. The dynamic cross-correlation matrices (DCCM) analysis investigates the correlated motions of amino acids that influence the dynamics of a protein (Kasahara et al., 2014). Bio3D suite (Grant et al., 2006) was used for DCCM analysis through which correlation motion was estimated by the projection of covariance matrix σ between 2 C_α_ atoms i and j of proteins applying the following equation:

$$\sigma_{ij}=\frac{\langle \Delta r_{i}\left(t\right)\cdot \Delta r_{j}\left(t\right)\rangle }{\sqrt{\langle {\left\|\Delta r_{i}\left(t\right)\right\|}^{2}\rangle \sqrt{\langle {\left\|\Delta r_{j}\left(t\right)\right\|}^{2}\rangle }}}.$$ (1)

In the above equation, r_i_(t) defines the projected vector of atom i as a function of time, whereas ensemble over average time is represented by ⟨“⟩ and change in position of C_α_ atoms, i and j with respect to their original position at a given time that is symbolized as ∆r_i_(t) and ∆r_j_(t). Correlation movements that were projected *via* estimation of matrices were then subsequently visualized through two-dimensional cross-correlation maps. The variation in correlative motion on these dynamics cross-correlation maps was interpreted in terms of correlation (positive) and anti-correlation (negative) movements with respect to color appeared over maps. The last 40 ns from each trajectory were used for DCCM analysis.

### Principal Component Analysis (PCA)

PCA is able to identify dominant motions and maximize variation during protein flexibility (Balsera et al., 1996). The results are presented as variations in the values of a small number of collective coordinates. The Cα coordinates were used as an input for PCA. More specifically, PCA was carried out to provide a quantitative and comparative analysis between the two states of the HCN hexamers. The dimensionality reduction was carried out using the PyPcazip program (Shkurti et al., 2016) and the porcupine plots were generated using in-house scripts (Haider et al., 2008). All the trajectories were combined so that all share the same subspace and comparisons can be made. A dot product matrix between the eigenvectors identified by the PCA on the Deoxy- and Oxy-HCN is calculated.

### Solvent Accessible Surface Area

Solvent accessible surface area (SASA) can be used as a tool to assess the relative changes in conformational dynamics of homo- and heterodimers or multimer unit proteins (Marsh and Teichmann, 2011). The experimental studies have reported that HCN can exist in two different states; open, before oxygenation, and closed after oxygen passes to the binding site. These acquired states are entirely dependent on the conformational integrity of the hexamer. Thus, an estimation of SASA allows us to observe changes in the conformation of individual subunits and the hexamer as a whole. SASA was calculated using the “gmx sasa” module as implemented in the GROMACS tools (Abraham et al., 2015).

### Interface Accessible Surface Area (IASA)

The interactions at the interface of the top and bottom trimers in the dimer-of-trimers were evaluated by means of estimation of interface size or interface area through calculating the subsequent buried accessible surface area (ASA) upon complex formation among interface amino acid residues of individual subunits (Chen et al., 2013). Similarly, interactions at the interface of two dimers of trimers in hexamer were assumed to affect the entire interfacial dynamical properties of the whole protein.

The effect of contacts on the inter-subunit dynamics and the associated dynamical behavior of the hexamer was assessed by calculating the accessible surface area that is buried as a result of inter-subunit contacts between dimer-of-trimer in both Deoxy- and Oxy-HCN *via* NACCESS (Hubbard and Thornton, 1993). Estimation of the interface accessible surface area (IASA) was carried in such a way that each trimer of the hexamer was assigned as two chains, A and B for which accessible surface area or change in the accessible area (∆ASA) after complex formation was separately computed for chain A (ASA_A_) and B (ASA_B_) with subsequently combined computation for both chains AB (ASA_AB_) as explained in the following equation:

$$\text{IASA}\;=\;{\text{ASA}}_{\text{ChainA}}\;+\;{\text{ASA}}_{\text{ChainB}}\;-\;{\text{ASA}}_{\text{AB}}.$$ (4)

The IASA was calculated over from 100 snapshots that were extracted from the last 40 ns from each replicate simulation.

### Solvent Cavity-Pocket Analysis

Water is hypothesized to play a major role in passing oxygen up to the bi-nuclear copper-containing active site *via* cavities or tunnels formed within or at the interface of subunits. Evaluation of solvent cavities was thus carried out for both Oxy and Deoxy-HCN forms of the hexamer. Pocket analysis was done by calculating their opening and closing frequency in each individual subunit and then subsequently for the complete hexamer. Next, the cavities with maximum frequency were sorted and their volumes calculated. 200 snapshots from the last 40 ns of each replicate were used for pocket detection. MDPOCKET suite was used for this analysis (Schmidtke et al., 2011).

### Tunnel Analysis

The Caver 3.03 beta software (Chovancova et al., 2012) was used for the effective analysis of tunnels in structures derived from the molecular dynamics trajectories. This version uses an additional Voronoi diagram and is useful for the analysis of large macromolecules. For caver analysis, 330 frames were extracted from the last 40 ns of the Oxy- and Deoxy-HCN trajectories. The oxygen atom in Oxy-HCN and copper atoms in the Deoxy-HCN were selected as the starting point for tunnel identification. The probe radius was set at 1.0 Å and the clustering threshold value was kept at 10.0 to evaluate cluster results. The tunnels were ranked based on their average bottleneck radius (Å) and throughput values. The bottleneck radius provides the maximal probe size which can fit in the narrowest part of the tunnel, while the throughput value reflects the probability that the pathway is used as a route for transport of the substances using the formula e^-cost^, where e is Euler’s number and the cost is a function defined as

$$\overset{L}{\underset{0}{\int }}r{\left(l\right)}^{-2}\;dl,$$

where *L* is the length of a path, *r(l)* is a function defining the radius of the largest ball which does not collide with the atoms of the structure and is centered at the point on the pathways axis in the distance *l* from the starting vertex (Stourac et al., 2019).

### Convolutional Variational Autoencoder

The convolutional variational autoencoder (or CVAE) was implemented for analyzing the simulation trajectories. The CVAE was previously used successfully for multiple different cases, from analyzing protein folding (Bhowmik et al., 2018) to enzyme dynamics (Romero et al., 2019; Akere et al., 2020), to recent COVID-19 related molecular mechanism studies (Acharya et al., 2020; Cho et al., 2021). This specially built CVAE on the HPC platform is able to cluster different microstates of any system based on their subtle difference among 3D structures influenced by various local or global motions (Yoginath et al., 2019).

**FIGURE 3:**
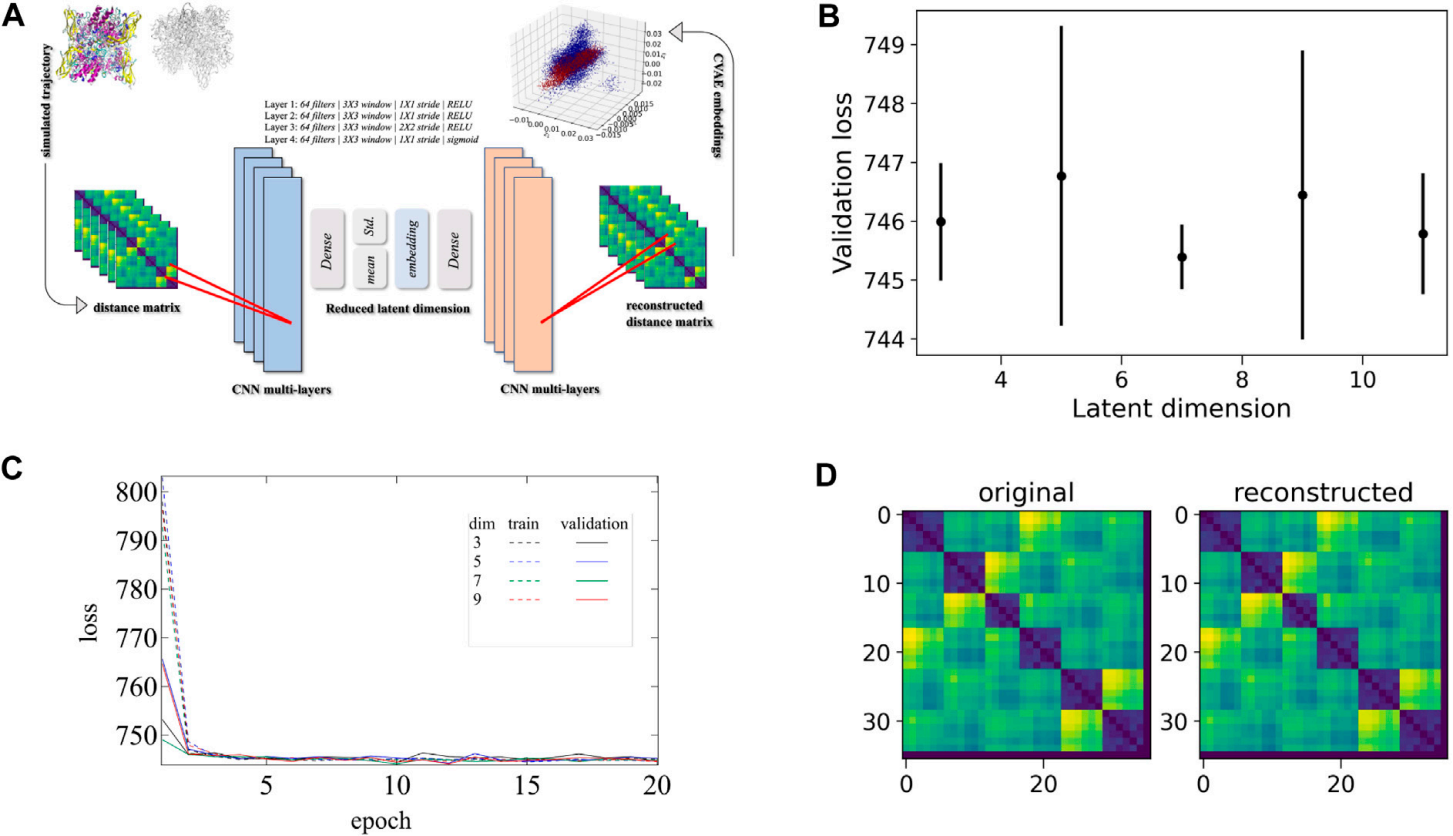
CVAE-based deep learning implementation. **(A)** The CVAE implementation where distance matrix is used as input data for generating the low-dimensional representations. The input data is initially trained where the quality of training is followed by the different loss values at different dimensions over epochs. **(B)** The validation loss is shown at different latent dimensions for determining the optimum values of the low dimension. **(C)** The training and validation loss are assessed over epochs at various dimensions. **(D)** Comparison between original input data and reconstructed data are shown to ensure no loss of essential information during the compression and reconstruction process

Essentially, the CVAE architecture is driven by a specific deep learning algorithm. Briefly, the CVAE is built on top of a traditional autoencoder with a variational approach. An autoencoder, in general, has an hourglass-like architecture where the high-dimensional input data is fed. Only the essential information is captured by the autoencoder as the high-dimensional data passes through it. The essential information is then used to reconstruct the original high-dimensional data to make sure there is no loss of information during the compression mechanism of the autoencoder. The added variational approach is a key component that optimizes the reduced dimension captured data and forces that to be distributed normally over the latent space, thus ensuring the efficient utilization of the latent space. Simultaneously this also helps the captured data not to be spreader sparsely and gain the useful knowledge to generate new conformations if required. In the end, the convolutional layers or CNN layers are included so that the local and global information is captured in an efficient way from the multi-layered complex biomolecular structures. The complete schematic CVAE architecture is shown in Figure 3A.

First, the distance matrix of the two systems (i.e., Oxy-HCN and Deoxy-HCN) was built separately using the 36 Histidine residues that coordinate with copper (Figure 2; His172, His177, His204 and His324, His328, His 364). With the parallelized version of the CVAE using the Horovod library, the input distance matrix was directly fed into the CVAE architecture. The training was performed on the Summit supercomputer with a fixed number of epochs. This number is determined by the trade-off between the convergence of loss value and variance bias. The training data were randomly divided into 80:20 ratio for training and validation dataset, respectively. The individual batch size was kept low so that the generalization gap for large-batch size training could be avoided. In order to select the optimal values for clustering parameters with the best reconstruction quality, the training was performed at various latent dimensions. Figure 3B shows how the loss function varies in different dimensions. Comparing the different loss values (Figure 3C) and loss values in different dimensions (Figure 3B) it is evident that dimension 7 is the best latent dimension to work with. The comparison between the original and reconstructed distance map of the system is also shown in Figure 3D.

## Results and Discussion

**FIGURE 4:**
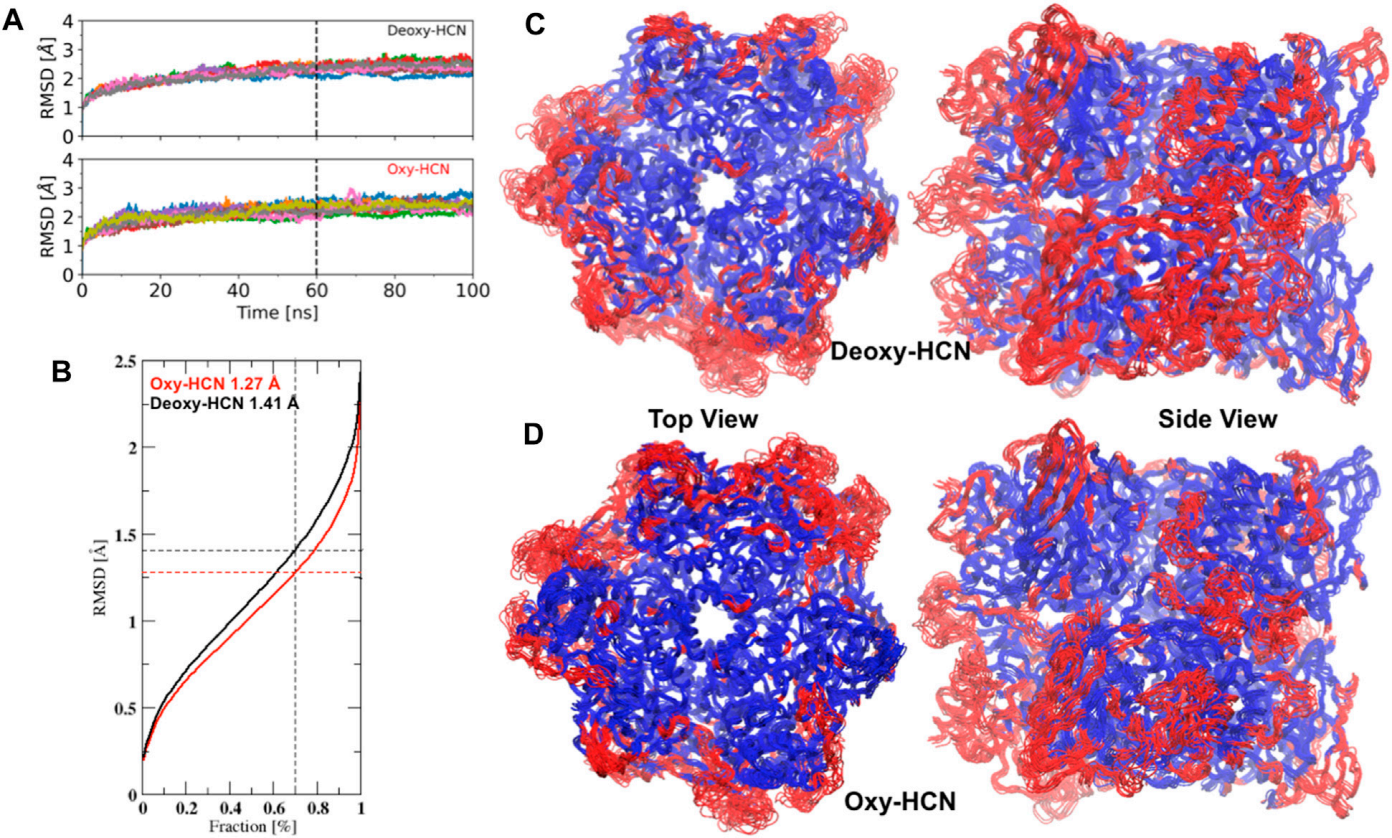
A comparison of Cα conformational drifts between the deoxygenated and oxygenated hemocyanin hexamers. **(A)** Average Cα RMSD for Deoxy-HCN (2.55Å ± 0.04) and Oxy-HCN (2.43Å ± 0.04) are similar when calculated over the last 40 ns equilibrated section of the trajectories. **(B)** 70% of Cα atoms in the simulation can be aligned to below 1.41 Å in Deoxy-HCN (black) and 1.27 Å in Oxy-HCN (red). Top and side views of **(C)** Oxy-HCN and **(D)** Deoxy-HCN structures, highlighting the least mobile Cα atoms (70%) that form the core (blue). The regions of the structure, which are more mobile (red) include the surface loops or short loops that connect different secondary structure elements together.

The conformational drift of both systems, Deoxy-HCN and Oxy-HCN, were evaluated by calculating the root mean square deviation (RMSD). Conventional methods calculate RMSD by aligning the structure in each frame of the trajectory to a reference structure. This rigid body alignment has a drawback, in the sense that if a small region of the structure is highly flexible, then the RMSD increases for all atoms. This results in an incorrect quantification of the structural deviations and thus poorly assesses important motions associated with biological function. As seen in the case of HCNs, conventional RMSD is unable to differentiate between the dynamics of the two states. The average Cα RMSD for Deoxy-HCN (2.55Å ± 0.04) and Oxy-HCN (2.43Å ± 0.04) are very similar when calculated over the last 40 ns of the trajectories (Figure 4A). To overcome this caveat, RMSD was calculated using the MDLovoFit algorithm. This method aligns a fraction of the structure displaying the smallest displacements and enables mobile structures to be identified. It was possible to align 70% of Deoxy-HCN to below 1.41 Å and Oxy-HCN to 1.27 Å (Figure 4B). The regions of low mobility (blue) are the core of the protein, while the surface loops and loops connecting secondary structure elements contribute to regions of high (red) mobility (Figures 4C,D). The lower RMSD in Oxy-HCN was reasoned to be a result of the collapsed cavities or tunnels due to enhanced inter- and intra-subunit interactions that stabilized the oxygenated state. This is consistent with the experimental finding that the oxygenation process enhances the stability of the hexamer (Sterner et al., 1995; Bux et al., 2018).

**FIGURE 5:**
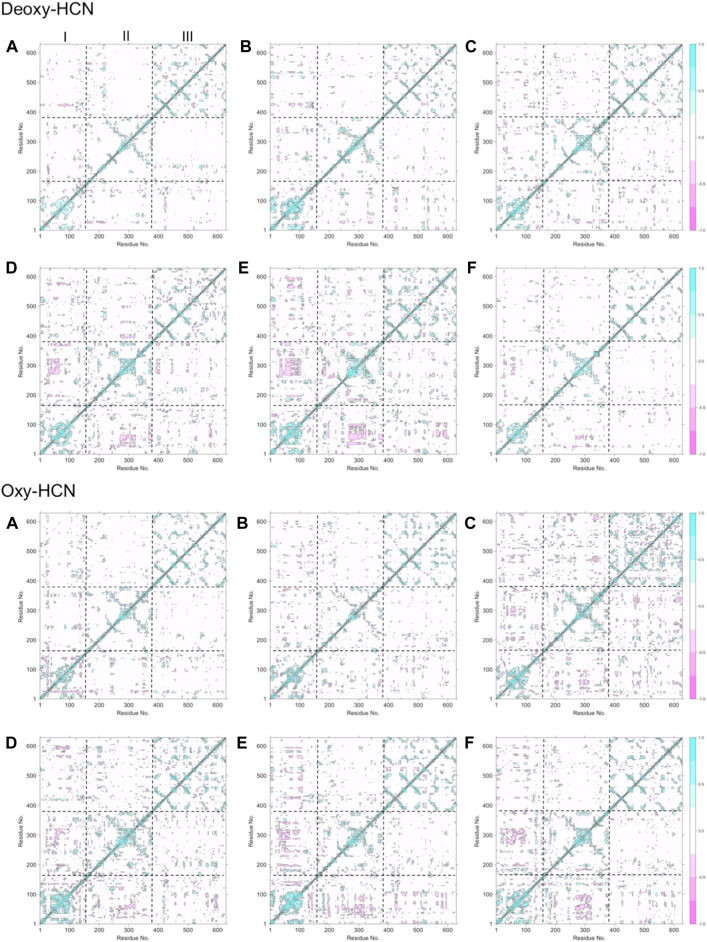
Dynamic cross-correlation matrix (DCCM) maps depicting cross-correlated motion for all six subunits in **(A)** Deoxy-HCN and **(B)** Oxy-HCN hexamers. Boundaries of domains I, II, and III are represented by dotted lines. Domains III and I predominantly show positively correlated motions.

The flexibility of both systems was assessed by calculating the root mean square fluctuations (RMSF) of Cα atoms of protein from the last 40 ns of the trajectories (Supplementary Figure S1). Each subunit within the HCN hexamer showed comparable flexibility in both forms of Deoxy- and Oxy-HCN states. Each monomeric unit consists of three domains: domain I comprising amino acid residues from 1 to 154, II that ranges from 155 to 380, and III that contains amino acid residues from 381 to 628. Of these, domains III and I were found to show more flexibility as compared with domain II in Oxy-HCN. The lower flexibility of domain II was reasoned to be due to the presence of oxygen in the metal binding site within this domain and also due to its spatial position, being sandwiched between domains III and I. In Deoxy-HCN dynamics, three subunits showed an increase in flexibility in domain II, suggesting that residues can be flexible in the absence of oxygen. This observation warranted further investigation into the correlated motions of each domain within the subunits. This was assessed by means of estimating correlated motions across each domain of the subunits individually along with the collective approximation of the individual subunits in the hexameric protein for both Deoxy-HCN and Oxy-HCN systems (Figure 5).

The dynamic cross-correlation maps between Deoxy-HCN and Oxy-HCN are comparable (Figure 5). Domains III and I in each subunit displayed positive correlated motions and smaller anti-correlated motions. Domain II in each subunit displayed more anti-correlated motions. The observations from the dynamic cross-correlation analysis highlight the relationship between oxygenation and associated changes observed between the three domains in the hexameric protein. This is suggestive of the dynamic equilibrium between different conformational states that makes the passage of oxygen possible in each subunit within the hexamer.

**FIGURE 6:**
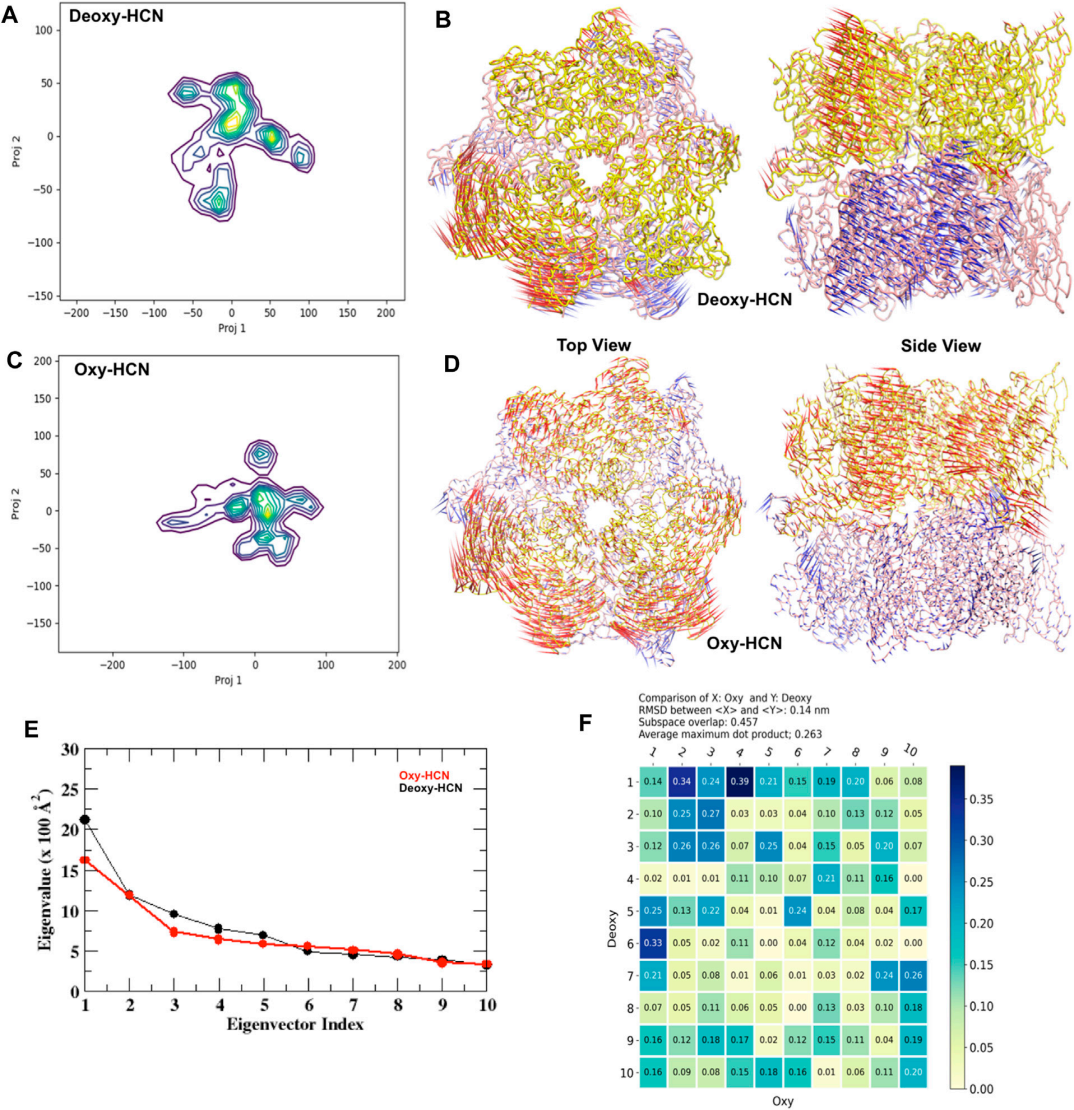
Principal component analysis (PCA). **(A)** Projections 1 and 2 from Deoxy-HCN represented as a heat map. **(B)** Top and side view of principal component 1 from Deoxy-HCN. **(C)** Projections 1 and 2 from Oxy-HCN represented as a heat map. **(D)** Top and side view of principal component 1 from Deoxy-HCN. **(E)** A scree plot comparing the eigenvalue versus eigenvector index in the two systems. **(F)** A subspace analysis between Deoxy-HCN and Oxy-HCN.

Further quantification into the similarities and differences in the global motion of the hexameric protein was assessed using principal component analysis (PCA). PCA was able to extract and filter dominant motions from a set of sampled conformations and define their respective essential space. The top mode was analyzed and is illustrated as a porcupine plot (Figure 6).

The dominant mode (PC1) in Deoxy-HCN is the rotation of the top trimeric ring with respect to the bottom. The three subunits in the top ring move clockwise and the bottom three anticlockwise. The PC1 in Oxy-HCN is dominated by a clam-shell-like movement between two adjacent subunits in each ring (Figure 6). A pairwise comparison between the Deoxy-HCN and Oxy-HCN states was made by calculating the dot product matrix between the eigenvectors identified from PCA of their corresponding states. Such comparison allows quantitative assessment of similarity between the dynamics in the two different systems. Deoxy-HCN and Oxy-HCN simulations have a subspace overlap of ∼45.7% and an average maximum dot product of 0.26. The most significant similarity observed between Deoxy-HCN and Oxy-HCN is when comparing PC1 of Deoxy-HCN and PC4 of Oxy-HCN, with an inner dot product of 0.39 (Figure 6). The motion in this PC is dominated by loop interactions at the interface of the two concentric rings. The presented analysis suggests that the Deoxy-HCN and Oxy-HCN simulations occupy only ∼50% of the same conformational (essential dynamics) subspace, though not always sampling the same regions in that space. Such observations from PCA also supported the assumption regarding large-scale dynamic changes when the proteins move from a deoxygenated state to a more stable oxygenated state. Moreover, the variation in the motion of the Deoxy-HCN and Oxy-HCN systems observed in PCA also indicated that the inter-residue contacts at the interface of each subunit might play an important role in the dynamics of the hexamer.

**FIGURE 7:**
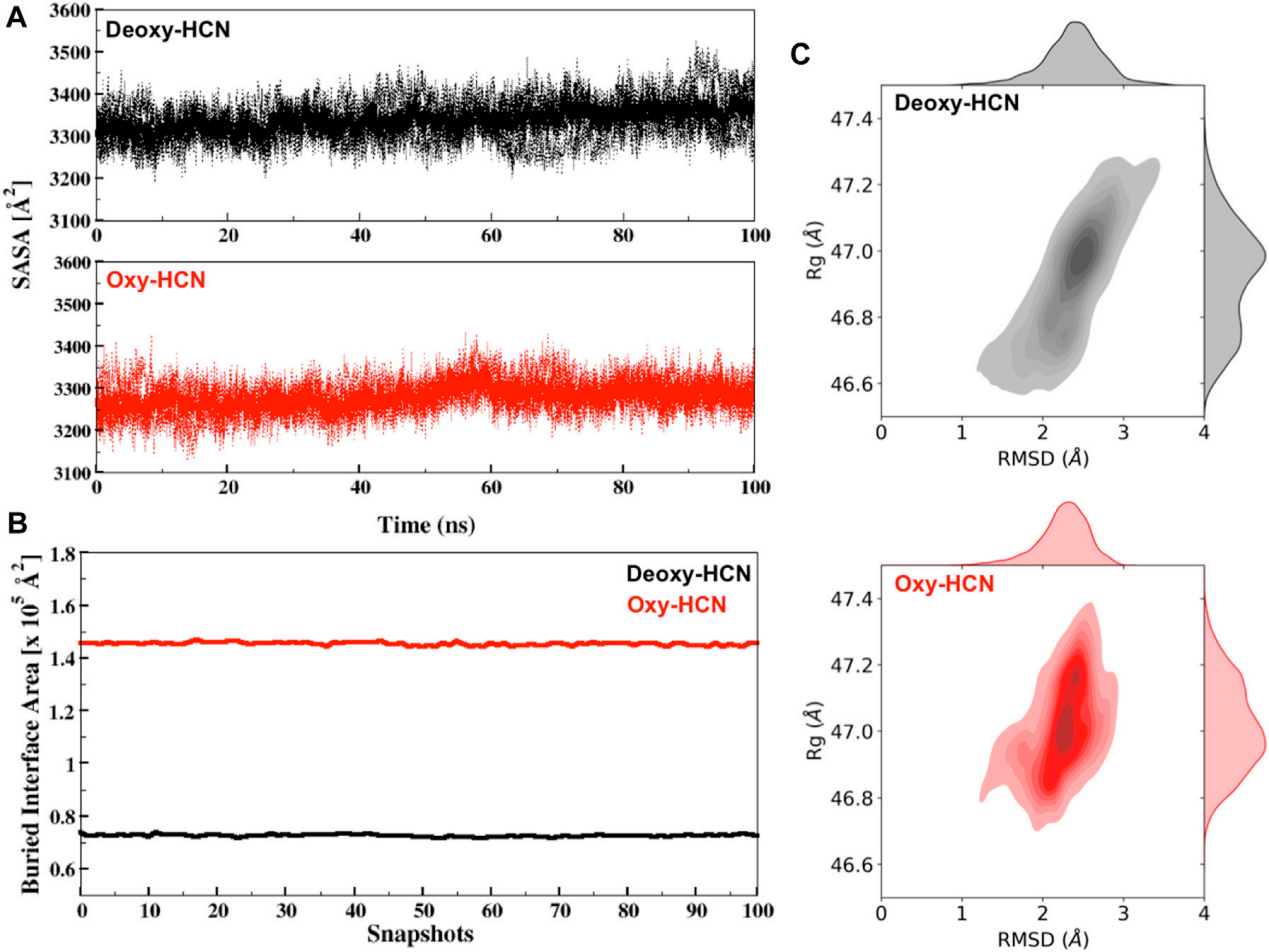
**(A)** Solvent accessible surface area (SASA) for Deoxy-HCN (black) and Oxy-HCN (red) of all subunits (dashed) and their average (bold). **(B)** Interface accessible surface area (IASA) buried due to inter-residual contacts for Deoxy-HCN (black) and Oxy-HCN (red) of 100 snapshots extracted from the last 40 ns of each trajectory. **(C)** The radius of gyration versus Cα-RMSD correlation plot for Deoxy-HCN (black) and Oxy-HCN (red).

The dynamic states of the hexamer were then assessed by calculating the solvent-accessible surface area (SASA) of each subunit and then averaging it over the hexamer. The changes in SASA were then correlated with the motions to highlight variation in structural dynamics. Deoxy-HCN showed a relatively larger solvent accessible surface area (3337.48 Å^2^ ± 21.3) as compared to Oxy-HCN (3280 ± 19.9 Å^2^) hexamer (Figure 7A). The differences between SASA for the two states are very similar to draw any conclusive inferences regarding the dynamics of the systems.

We next analyzed the radius of gyration (Rg) of the hexamer and correlated it with the RMSD (Figure 7C). Rg allows the estimation of the compactness of each system. The Rg values for the two systems are very similar (Deoxy-HCN 47.2Å ± 0.13; Oxy-HCN 46.9Å ± 0.16) and the global compactness of the systems is indistinguishable between the two states of the hexameric protein.

To assess interfacial flexibility, the interface area was quantified by measuring the buried interface accessible surface (IASA) that became concealed or buried as a result of interactions taking place among interfacial residues of two trimeric rings of dimer in hexamer (Figure 7B). Oxy-HCN was observed to have relatively larger buried interface area (1.45 × 10^5^ Å^2^ ± 591.9) as compared to Deoxy-HCN (0.72 × 10^5^ Å^2^ ± 401.6). A lower value of buried interface in case of Deoxy-HCN suggested more flexibility, which increased the chances of inter residue contacts taking place at the interface of dimer of trimers. In case of Oxy-HCN, a higher value of the buried interface was consistent with the diminished flexibility at the interface and suggested that the dense contacts were a part of the accessible surface area that became concealed or buried in the oxygenated state.

**FIGURE 8:**
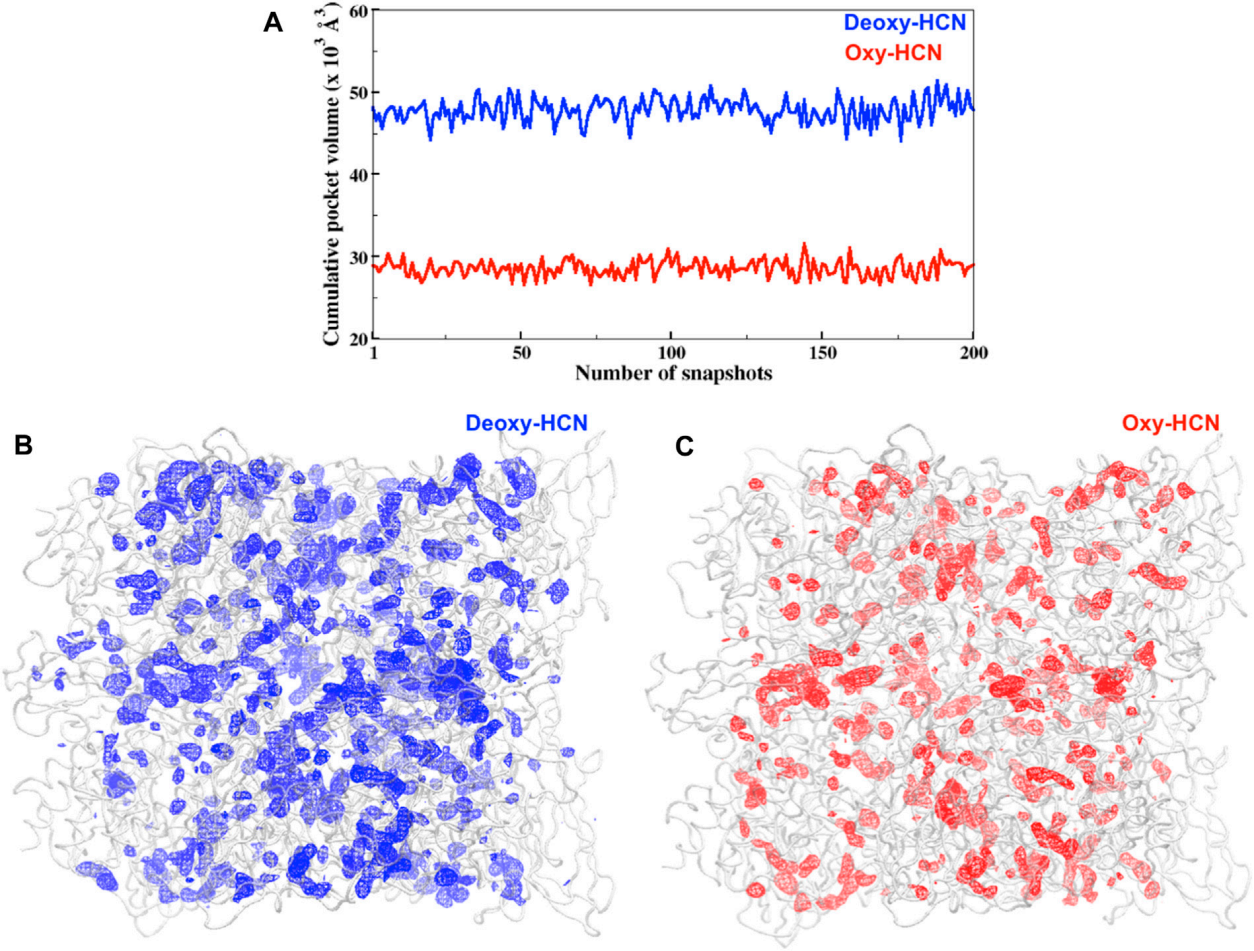
**(A)** Cumulative pocket volumes of cavities facilitating the passage of oxygen within subunits for Deoxy-HCN (blue) and Oxy-HCN (red). The volume was calculated from 200 snapshots of each system, which was extracted from the last 40 ns of each trajectory **(B)** Three-dimensional depiction of cavity density (mesh) spread in the (B) Deoxy-HCN (blue) and **(C)** Oxy-HCN (red) hexamers. The lower cumulative pocket volume Oxy-HCN (red) indicates a more stable closed state in Oxy-HCN.

Hemocyanins have been reported to have solvent cavities or channels within and at the interface of each subunit (Hazes et al., 1993; Magnus et al., 1994). Thus, investigation of these tunnels or cavities was also carried out to understand their behavior in their respective dynamic states (Figure 8). The cumulative pocket volume calculated in Deoxy-HCN (47.8 × 10^3^ Å^3^) was greater than that in Oxy-HCN (28.5 × 10^3^ Å^3^) (Figure 8).

**FIGURE 9:**
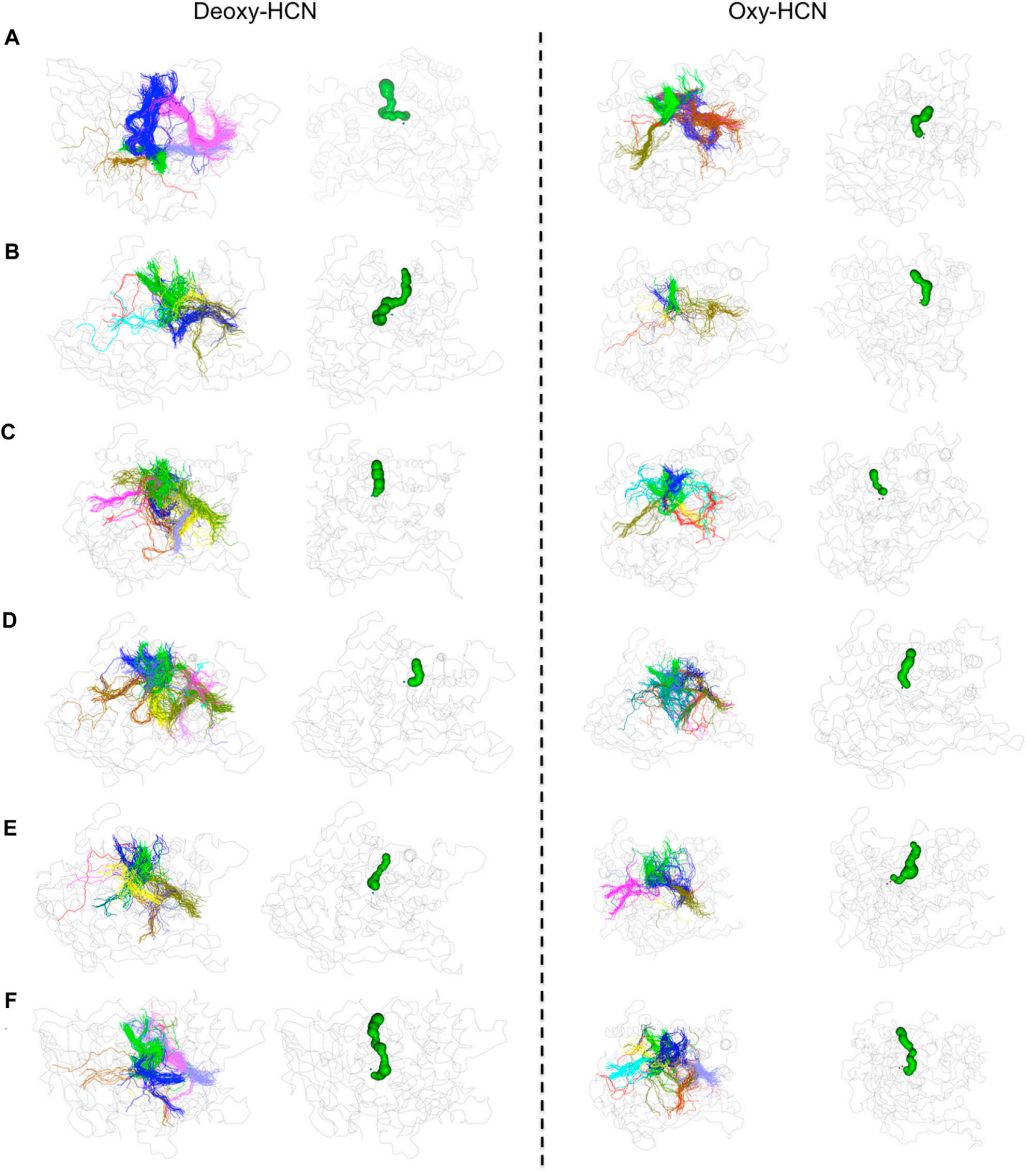
Tunnel analysis in Deoxy-HCN and Oxy-HCN. Tunnel clusters identified in each monomer are illustrated with distinct colors. The tunnel formed by the top cluster is highlighted in green.

To further validate the observations drawn from the cumulative volume of cavities, dynamic tunnel formation was measured in the simulated systems. A summary of all clusters is tabulated in Table 1. Clusters were calculated for individual subunits within the hexamer and compared between the two states. The identified tunnel clusters are illustrated in Figure 9. The top cluster is the most important due to the higher throughput value and average bottleneck radius. The residues involved in tunnel formation for the top cluster are listed in Table 2. In Deoxy-HCN, 58 cumulative clusters were identified while in Oxy-HCN 54, clusters were observed. For each tunnel cluster, all residues were at least present in one snapshot and within 2 Å from an individual tunnel cluster. The dynamic properties of the tunnel were assessed using a bottleneck analysis. A bottleneck is defined as the narrowest part of the tunnel and the average ranged between 1.1 and 1.2 Å in Oxy-HCN. During the course of the dynamics, the constriction formed by residues expands from 1.0 Å to 1.8 Å, which is enough to allow the oxygen molecule to pass. Similarly in Deoxy-HCN, the bottleneck radius ranged from 1.0 to 1.3 Å, while the constriction is able to expand to 1.9 Å. In both systems, the top tunnel clusters identified within each monomer share several common residues. Of these, S182, E309, and N325 appear around the bottleneck and contribute towards the constriction of the tunnel. The relatively greater flexibility observed in Deoxy-HCN is in agreement with the conformational state that was ready to facilitate oxygen entry into the protein. Additionally, the cavities and tunnels in the Deoxy-HCN state are correspondingly spread across the interface of all subunits. Smaller and less dense cavities in Oxy-HCN are representative of the conformation of the protein that is acquired after oxygen has passed to the binuclear active site of the protein.

**TABLE 1:** Dynamic tunnel properties of Deoxy- and Oxy-HCN identified by Caver, using a probe radius of 1.0 Å and a clustering threshold of 10.0 (BR: bottleneck radius; TP: throughput).

Oxy-HCN						
-	**A**	**B**	**C**	**D**	**E**	**F**
**No. of clusters**	6	9	8	11	8	12
**No. of snapshots**	254	37	135	141	111	225
**Average BR (Å)**	1.20	1.10	1.24	1.09	1.14	1.19
**Max BR (Å)**	1.50	1.31	1.82	1.72	1.54	1.71
**Average TP**	0.50	0.48	0.56	0.51	0.48	0.40
**Deoxy-HCN**						
-	**A**	**B**	**C**	**D**	**E**	**F**
**No. of clusters**	7	6	10	14	10	11
**No. of snapshots**	331	101	204	145	95	133
**Average BR (Å)**	1.33	1.14	1.21	1.20	1.20	1.15
**Max BR (Å)**	1.93	1.60	1.71	1.73	1.72	1.60
**Average TP**	0.60	0.37	0.52	0.53	0.49	0.40

**TABLE 2:** Tunnel lining residues of the tunnel cluster with the highest average throughput and average bottleneck radius.

	Oxy-HCN
**A**	C48, F49, P51, L54, F91, W176, Y180, P181, **S182**, T183, K196, F200, **E309**, S310, N322, **N325**, T351, F360
B	C48, F49, P51, L54, **S182,** T183, F200, V308, **E309**, S310, S311, Y312, N322, L323, **N325**, W326, G327, M348, S354, F360, W363
C	F49, L54, W176, **S182**, F200, V308, **E309**, S310, S311, Y312, N322, N325, T351
D	C48, F49, H50, P51, L54, H175, W176, **S182**, T183, F200, **E309**, S310, S311, Y312, N322, **N325**, T351, S354, F360
E	C48, F49, P51, L54, E55, A57, R58, Y61, F91, W174, H175, W176, L178, V179, Y180, P181, **S182**, T183, W184, K196, F200, **E309**, **N325**, D350, T351, S354, F360
F	C48, F49, W176, Y180, P181, **S182**, R195, K196, G197, E198, L199, F200, M203, D302, I303, G305, A306, **E309**, S311, E313, D350, T351, S354, L355, F360
**Deoxy-HCN**	
A	F46, C48, F49, H50, P51, D52, L54, E55, F91, W176, L178, Y180, P181, **S182**, T183, K196, F200, **E309**, S310, S311, Y312, H317, G321, N322, **N325**, T351, F360
B	F46, S47, C48, F49, H53, L54, E55, A57, R58, H59, Y61, L88, F91, V95, A171, H172, W174, H175, W176, L178, Y180, P181, **S182**, T183, K196, F200, M203, M207, **E309**, **N325,** T351, S354, L355, F360, W363
C	S47, C48, F49, H50, P51, L54, W176, **S182**, T183, **E309**, S310, S311, Y312, H317, N322, S349, D350, T351, S352
D	S47, C48, F49, H50, L54, W174, W176, L178, **S182**, T183, F200, M203, **E309**, S310, S311, N322, **N325**, D350, T351, S352
E	C48, F49, H50, P51, L54, **S182**, T183, F200, M203, **E309,** S310, S311, N322, **N325**, D350, T351, S354, L355, F360
F	F49, H172, W176, V179, Y180, P181, **S182**, K192, K193, D194, R195, K196, G197, L199, F200, M203, M207, **E309**, S310, T351, L355, F360, A585, D587, H588, K589, Y590, M596

**FIGURE 10:**
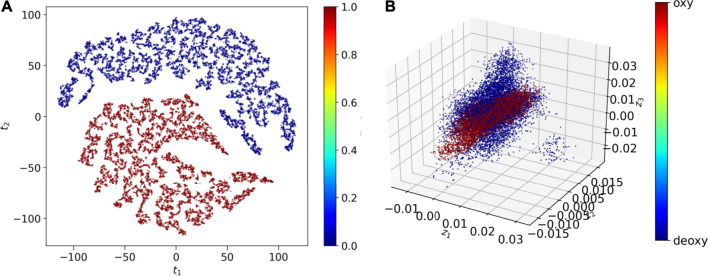
CVAE-based deep learning analysis. The reduced dimensional latent space of CVAE-learnt features of the high dimensional original input are shown in **(A)** 2D representation and **(B)** 3D representation following t-sne treatment on the compressed data. The results show that the two systems [i.e., Oxy-HCN (red) and Deoxy-HCN (red)] are different based on their local and global conformational dynamics.

The global dynamics of the Oxy-HCN and Deoxy-HCN are indistinguishable using conventional analysis like RMSD, radius of gyration, and solvent accessible surface area. Thus, a highly sensitive convolutional variational autoencoder (CVAE) based deep learning method was used to differentiate between the dynamics of the two systems. The CVAE model quality was evaluated in different latent dimensions. Dimension 7 turned out to be the best dimension to work with (Figure 3B). The loss behavior over epoch showed a normal trend in all dimensions but the value with the variation in the loss at dimension 7 was better than others (Figure 3B). This is because initially as the latent dimension increases, the model starts compressing less and thus acquires more representation ability. But gradually when the dimensions become too large, then the model might overfit while introducing additional noises. The overall loss attains an optimal value between the two extremes. For Oxy- and Deoxy-HCN systems, the CVAE model is quite stable, as the loss stays close to each other in different dimensions. The data was visualized using 2D-tsne (t-distributed stochastic neighbor embedding) (Figure 10A). CVAE is able to cluster the two systems (i.e., Oxy-HCN (red) and Deoxy-HCN (blue) distinctly based on their local and global conformational dynamics. This is visible both in 2D- and 3D-dimensional representation (Figure 10B). These results indicate that the dynamics of the oxygenated and deoxygenated systems are discernible.

## Conclusion

Molecular dynamics simulation was used to study the differences between the deoxygenated and oxygenated forms of hemocyanin hexamers. This 3768 residue system was assessed over a cumulative sampling time of around 1 µs (10 replicates of 100 ns each). Conventional structural analysis methods were unable to differentiate between the dynamics of the two systems. This is because the conformations of the starting structure of Oxy-HCN and Deoxy-HCN are very similar. Further investigation of the systems using PCA highlighted less than ∼46% subspace overlap. The most dominant motions identified the hexameric HCN system to function as dimer-of-trimers. The evaluation of interfacial dynamics by estimating the contacts between residues at the interface indicated that the Deoxy-HCN subunits experienced fewer contacts as compared with Oxy-HCN subunits. Calculations of the interface area *via* IASA further revealed that the residues at the interface of subunits Deoxy-HCN were less buried than in Oxy-HCN. Estimating the solvent cavities in terms of densities, frequencies and their volumes also found Oxy-HCN to have contracted solvent cavities. The average bottleneck radius of the tunnels identified in the Deoxy-HCN was greater than in Oxy-HCN. The deep learning analysis carried out as a function of the residues that bind to copper in the active site indicates that the two systems are different based on their local and global conformational dynamics. These observations, when taken together confirm a conformation for Deoxy-HCN that is ready to accept and transport oxygen to metal binding sites. Once the oxygenation process is completed, the system becomes stable as represented by the Oxy-HCN conformation.

## Data Availability

The raw data supporting the conclusions of this article will be made available by the authors, without undue reservation, to any qualified researcher.
