# Supplementary material for: Inter-Subunit Dynamics Controls Tunnel Formation During the Oxygenation Process in Hemocyanin Hexamers
Source: Front Mol Biosci. 2021 Sep 15;8:710623. doi: 10.3389/fmolb.2021.710623 (PMC8479113; doi:10.3389/fmolb.2021.710623)
Supplement: Supplementary file 1 [file DataSheet1.docx]

**Supplementary Information**

Khair Bux,^1^ Xiayu Shen,^2^ Muhammad Tariq,^1^ Junqi Yin,^3^ Syed Tarique Moin,^1^ Debsindhu Bhowmik,^4^ and Shozeb Haider ^2,*^

^1^ Third World Center for Science and Technology, H.E.J. Research Institute of Chemistry, International Centre of Chemical and Biological Sciences, University of Karachi, Karachi, 75270 Pakistan

^2^ UCL School of Pharmacy, London WC1N 1AX, United Kingdom

^3^ Center for Computational Sciences, Oak Ridge National Laboratory, Oak Ridge, TN 37830, USA

^4^ Computer Sciences and Engineering Division, Oak Ridge National Laboratory, Oak Ridge, TN 37830, USA

* Corresponding author: Shozeb Haider

Email: [Shozeb.haider@ucl.ac.uk](mailto:Shozeb.haider@ucl.ac.uk)

ORCID: Shozeb Haider: 0000-0003-2650-2925


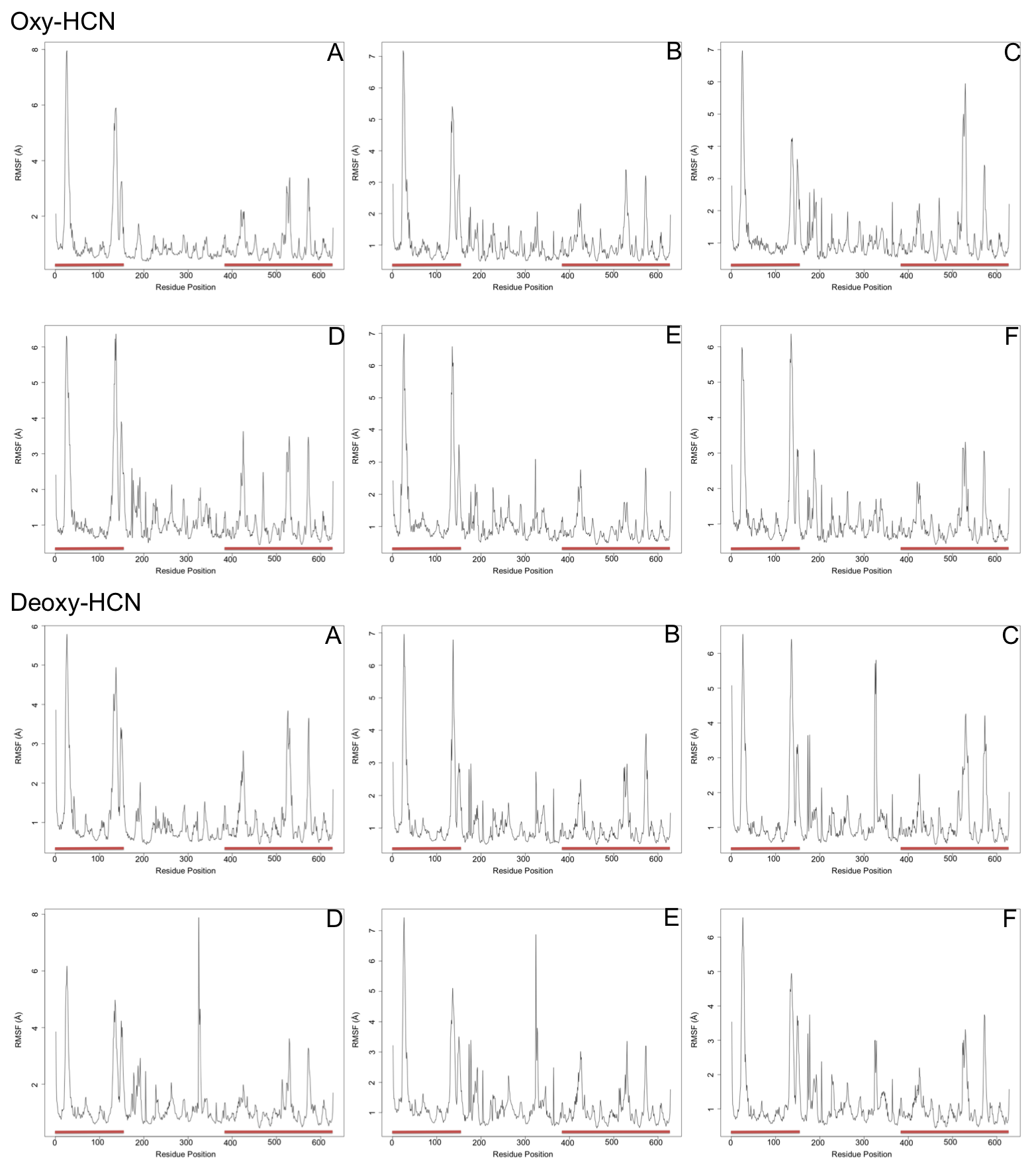


Figure S1: Root mean square fluctuation plots calculate for every subunit from the last 40 ns of the trajectories. The boundaries for domain III and I are indicated by the red line. In Oxy-HCN, domain III and I are largely flexible, where as domain II is stable. This could be because of the presence of oxygen bound in the metal centre in domain II. In Deoxy-HCN, domain II also displays fluctuations (in three subunits) along with domain III and I.
